# Understanding the Impact of Single-Helical Maize Amylose on Steamed Bun Hardness Enhancement

**DOI:** 10.3390/foods15101821

**Published:** 2026-05-21

**Authors:** Jiarui Yu, Zhihui Zhang, Shuai Ran, Xiaoxiao Li, Chunrui Wang, Junjie Guo, Xijun Lian

**Affiliations:** Tianjin Key Laboratory of Food Biotechnology, College of Biotechnology and Food Science, Tianjin University of Commerce, Tianjin 300134, China; yjr200109@126.com (J.Y.); zhangzhihui@tjcu.edu.cn (Z.Z.); ranshuai@tjcu.edu.cn (S.R.); lixiaoxiao@tjcu.edu.cn (X.L.); wangchunrui@tjcu.edu.cn (C.W.)

**Keywords:** salt-mediated co-crystallization, spectroscopic characterization, glutenin, ester band, hydrogen band, synergistic interactions

## Abstract

In this study, single-helical maize amylose (SHMAM) was successfully prepared via the sodium chloride-based eutectic solvent method. Incorporation of SHMAM into wheat flour for steamed buns significantly enhanced its hardness, with a 5% addition level yielding the maximum effect (hardness increased from 2318.7 ± 157.4 g to 3224.7 ± 98.1 g). Comprehensive structural characterization including FT-IR, XRD, DSC and ^13^C solid-state NMR revealed that during steaming hydrogen bonds formed between the C6 hydroxyl groups of SHMAM and sulfhydryl groups of Cys, α-amino groups of Lys, phenolic hydroxyl groups of Tyr, and ε-amino groups of Arg in glutenin. These interactions induced the conversion of β-sheets into α-helices and β-turns. As a result, a denser, more mechanically robust glutenin–starch network was formed, accompanied by a decreased water-holding capacity of glutenin and restricted interfacial water mobility between starch and glutenin phases. Collectively, these synergistic interactions enhanced dough compactness, stabilized the microstructural integrity of the dough matrix, and improved the hardness of the final steamed bun. This work establishes a novel, green, and scalable strategy for precisely modulating steamed bun texture, with broad implications for quality optimization in traditional wheat-based foods and potential benefits for dietary health.

## 1. Introduction

Starch, the most abundant natural storage polysaccharide, is ubiquitously present in major staple crops—including wheat, maize, rice, and sweet potato [1,2,3,4]. Owing to its natural abundance, economic viability, biodegradability, and physiological safety, starch serves as a fundamental raw material across both the global food industry and diverse industrial applications [5,6]. A substantial body of research has established that starch exerts profound effects on the critical functional properties of food systems, notably water-holding capacity [7], emulsification performance [8], rheological behavior [9], viscoelasticity [10], colloidal and thermal stability [11], gelation characteristics [12], and digestibility [13].

Steamed buns, a traditional fermented staple food widely consumed in northern China, are primarily prepared using wheat flour via controlled fermentation and steam cooking. Their popularity stems from operational simplicity, practicality, and economic affordability [14]. The textural quality of steamed buns plays a crucial role in determining their overall acceptability and consumer preference. Inadequate hardness compromises structural integrity, leading to surface collapse, excessive stickiness, and diminished chewiness [15,16]. Consequently, achieving optimal textural attributes—including a uniform surface appearance, balanced hardness, sufficient elasticity, and favorable mouthfeel—necessitates strategic enhancement of raw material quality, particularly wheat flour functionality. Importantly, appropriate hardness not only improves sensory performance but may also attenuate starch digestibility, thereby enhancing satiety, reducing postprandial glycemic response, and supporting long-term energy balance and body weight regulation [17,18].

During the thermal processing of starch-based products, the intra- and inter-molecular hydrogen bonds within starch chains are progressively disrupted. This disruption exposes free hydroxyl groups on glucan chains, facilitating water absorption and subsequent granule swelling. Ultimately, granular integrity is compromised, resulting in the concurrent leaching of amylose and amylopectin [19,20]. Amylose architecture—particularly its chain length distribution and conformational stability—is a critical determinant of starch digestibility. Recent work by Yan further demonstrates that thermally resilient single-helical amylose acts as an intrinsic cross-linking motif within the starch gel network [21]. By restricting amylopectin chain mobility during cooling and storage, such helices augment gel mechanical integrity, significantly improving hardness, elasticity, and chewiness in processed foods. In addition, endogenous wheat proteins modulate starch functionality through multiple non-covalent and competitive interactions—including hydrogen bonding, hydrophobic association, covalent cross-linking (e.g., disulfide bridges), and water competition—thereby altering starch morphology, pasting behavior, in vitro digestibility, and nutritional bioavailability [22,23].

Maize, one of the world’s most widely cultivated cereal crops, functions as both a primary dietary staple for human consumption and a high-nutrient feed source for livestock [24,25]. Maize starch is also extensively utilized across the food industry and diverse industrial sectors [26,27]. Recent findings indicate that even low concentrations of maize starch can effectively modulate dough’s rheological properties, particularly viscosity [28]. Further advances have shown that salt-mediated co-crystallization techniques enable precise modulation of starch molecular weight and amylopectin/amylose chain length distribution, leading to tailored physicochemical functionality [29]. Building upon these insights, our research group has developed a NaCl-based co-crystallization methodology grounded in salting-out effects and ion-mediated molecular assembly, which facilitates the scalable preparation of SHMAM with markedly improved molecular weight homogeneity [30]. Typically, steamed buns made from high-gluten flour exhibit a soft texture and relatively poor chewiness [31]. However, few studies have investigated the incorporation of homogeneous SHMAM to synergistically interact with endogenous components in high-gluten wheat flour, thereby reinforcing the dough network and enhancing the hardness and chewiness of steamed buns.

In this study, structural and thermal characterization was performed using high-performance size-exclusion chromatography (HPSEC), Fourier-transform infrared spectroscopy (FTIR), solid-state ^13^C nuclear magnetic resonance (NMR), X-ray diffraction (XRD), and differential scanning calorimetry (DSC). This work establishes SHMAM as a novel, sustainable functional ingredient for precise textural modulation in steamed bun formulations, specifically achieved by regulating hardness to enhance overall product quality attributes.

## 2. Materials and Methods

### 2.1. Materials

Common maize starch was procured from He Nan Enmiao Food Co., Ltd. (Zhengzhou, China). Enzyme preparations including amylase, Microbial lipase and protease were purchased from Beijing Solarbio Science & Technology Co., Ltd. (Beijing, China). High-gluten wheat flour was sourced from Wudeli Flour Mill Co., Ltd. (Handan, China). All other chemical reagents involved were acquired from Tianjin Fengchuan Chemical Reagents Co., Ltd. (Tianjin, China).

### 2.2. Extraction of Amylose

Maize amylose was obtained according to the reported method [28] with some modifications. Common maize starch was dispersed in deionized water to yield a 20% (*w*/*v*) suspension, which was then heated to 70 °C under magnetic stirring until complete granular swelling was achieved. The resulting suspension was cooled to the ambient temperature and subsequently frozen at −20 °C for 16 h. After thawing, the sample was re-gelatinized at 90 °C until optical transparency was attained, followed by autoclaving at 120 °C for 20 min. The autoclaved solution was cooled to room temperature and stored at 4 °C for 24 h to induce retrogradation. Sequential enzymatic hydrolysis was carried out as follows: (i) liquefaction with thermostable α-amylase (3% *w*/*w*, relative to starch mass) at 90 °C until complete viscosity reduction; (ii) proteolytic digestion with microbial protease (0.1% *w*/*w*) at 55 °C for 24 h; and (iii) lipolytic hydrolysis with microbial lipase (0.1% *w*/*w*) at 45 °C for 24 h. The enzymatic hydrolysate was washed three times with deionized water by centrifugation. Purification was performed by dissolving the residue in 2 mol/L NaOH, neutralizing the alkaline solution with dilute HCl to pH ~7, and precipitating amylose with cold ethanol (*v*/*v*, 3:1 ethanol-to-solution ratio). The precipitate was recovered by centrifugation, washed three times with deionized water, and lyophilized to afford purified maize amylose.

### 2.3. Co-Crystallization for SHMAM

The co-crystallization procedure was conducted following the established method [30]. Maize amylose was subjected to eutectic co-crystallization under systematically controlled conditions. Starch suspensions were prepared using a fixed volume-to-mass ratio of 20:1 (*v*/*w*) of 0.5% (*w*/*v*) NaCl aqueous solution to native starch. The mixtures were incubated at 4 °C for 24 h to facilitate eutectic co-crystallization. Formation of single-helical amylose–metal complexes was monitored periodically via iodine binding assays, with completion indicated by the complete disappearance of the characteristic blue–black iodine–amylose complex color. Following incubation, SHMAM precipitates were recovered by centrifugation (10,000× *g*, 15 min), washed repeatedly with deionized water until the supernatant yielded no turbidity upon addition of AgNO_3_ solution—confirming complete removal of chloride ions. The purified product was then lyophilized to ensure complete dehydration and stored at 4 °C pending further characterization or application.

### 2.4. Preparation of Steamed Buns and Hardness Measurement

SHMAM was incorporated into wheat flour at substitution levels of 0%, 1%, 5%, and 10% (*w*/*w*, on a starch-to-flour basis). Active dry yeast was rehydrated in a minimal volume of warm water (35–37 °C) prior to incorporation, and added to the flour blend at a ratio of 1:100 (*w*/*w*, yeast-to-flour). Water was gradually added during mixing until a cohesive, flocculent dough mass was obtained. The dough was then mixed in a laboratory-scale dough mixer for 10 min, followed by two sequential fermentation stages under controlled conditions (35 °C, 85% relative humidity). Thereafter, dough pieces weighing 20 g each were manually shaped into uniform spheres. The buns were steamed for 20 min at 100 °C under atmospheric pressure, cooled to ambient temperature (25 ± 2 °C), and subsequently stored at 4 °C for 0, 1, 3, and 6 days to evaluate storage stability.

Texture analysis was conducted using a TA.XT Plus Texture Analyzer (Stable Micro Systems, Surrey, UK), fitted with a cylindrical P/18 probe, following the standardized Texture Profile Analysis (TPA) protocol described by Aleixandre et al. [32]. The test parameters were set as follows: pre-test, test, and post-test speed of 5 mm/s; two consecutive compressions to 40% strain; a 5 s dwell time between compressions; and a trigger force of 5 g. For each sample, three independent replicates were performed, and the mean values were reported.

### 2.5. UV Determination

Referring to the method of He et al. [30], the absorbance of the SHMAM solution was measured by UV-2501PC UV-VIS spectrophotometer (Shimadzu, Kyoto, Japan). Firstly, an appropriate amount of SHMAM sample was dispersed in a 2.0 M NaOH solution under magnetic stirring and adjusted to neutrality with a 6 M HCl solution to ensure the accuracy of the measurement. Subsequently, 2 mL of 5% (*m*/*v*) I_2_/KI solution, prepared by dissolving 0.625 g of I_2_ and 1.875 g of KI in 50 mL of distilled water, was added to the SHMAM sample solution. The absorption spectrum of the sample within the 250–700 nm wavelength range was recorded using the UV spectrophotometer (UV-1800, Shimadzu, Kyoto, Japan).

### 2.6. Morphological Analysis

The morphology of the SHMAM samples was observed by CI-L NIKON microscope equipped with a Digital Sight Imaging system (CKX53, Olympus Co., Tokyo, Japan). Before measurement, a single drop of starch suspension was transferred onto a glass coverslip. Then, the images with various magnifications were collected, which could ensure the samples were observed in a fixed field.

### 2.7. Chain Length Distribution

High-performance anion-exchange chromatography coupled with pulsed amperometric detection (HPAEC-PAD; Dionex Corporation, Sunnyvale, CA, USA) was used to characterize the degree of polymerization (DP) distribution of starch samples. SHMAM (0.1 g) was dissolved in 50 mL of 4.0 M aqueous KOH and subsequently neutralized with 6.0 M HCl to pH ≈ 7.0. The resulting solution was incubated with pullulanase (0.5 U, 0.1 mL) at 45 °C for 24 h to achieve complete debranching. Enzymatic activity was terminated by heating the mixture to 100 °C for 10 min. The sample was then centrifuged at 20,000× *g* for 10 min at 4 °C, and the clear supernatant was filtered through a 0.5 μm syringe filter prior to HPAEC-PAD analysis.

### 2.8. Molecular Weight Determination

The molecular weight distributions of the test samples were determined using high-performance size-exclusion chromatography (HPSEC) coupled with multi-angle laser light scattering (MALLS) and refractive index (RI) detection. The system comprised a DAWN HELEOS II MALLS detector and an Optilab T-rEX RI detector (Wyatt Technology Corporation, Santa Barbara, CA, USA). Chromatographic separation was achieved on two Organic SEC columns (Styragel^®^ HMW 6E; DMF-compatible, 250 mm and 1000 mm, 7.8 mm × 300 mm each; Waters Corporation, Milford, MA, USA) connected in series. The mobile phase consisted of HPLC-grade dimethyl sulfoxide (DMSO) containing 50 mM sodium nitrate (NaNO_3_), filtered through a 0.22 μm PTFE membrane and degassed prior to use. Analyses were performed at 45 °C with a constant flow rate of 0.3 mL/min.

A 5 mg sample of starch was dispersed in 0.25 mL of 2.0 M aqueous NaOH solution. Subsequently, 2.0 mL of deionized water was added to facilitate complete solubilization, and the mixture was vortexed vigorously for 2 min to ensure homogeneity. The resulting solution was filtered through a 0.5 μm polyethersulfone (PES) membrane syringe filter prior to injection. Aliquots of 200 μL were manually injected into the high-performance size-exclusion chromatography (HPSEC) system equipped with mixed-bed columns (e.g., Shodex OHpak SB-806M HQ). Calibration was performed using dextran standards (T40 and T2000), and molecular weight distributions—including weight-average molecular weight (Mw) and number-average molecular weight (Mn)—were determined using Astra software (Version 5.3.4, Wyatt Technology).

### 2.9. Fourier-Transform Infrared Spectroscopy (FT-IR)

FTIR spectra were recorded on a PerkinElmer FTIR spectrometer over the wavenumber range of 4000–400 cm^−1^ with a spectral resolution of 4 cm^−1^. All samples were uniformly dispersed in spectroscopic-grade KBr and compressed into transparent pellets at a mass ratio of 1:100 (sample:KBr). Secondary structural components—namely, α-helix (1650–1660 cm^−1^), β-sheet (1600–1640 cm^−1^), β-turn (1660–1695 cm^−1^), and random coil (1640–1650 cm^−1^)—were quantified by deconvolution of the amide I band, following the methodology described by Xue et al. [33].

### 2.10. X-Ray Diffraction (XRD)

For the determination of XRD patterns, all the samples were exposed to the Cu Kα radiation (α = 1.54 Å) at 45 kV and 40 mA. The scanning region of the diffraction angle (2θ) was set from 3° to 50° with a step interval of 0.02° and a scan rate of 0.5°/min.

### 2.11. Differential Scanning Calorimetry (DSC)

DSC measurement was performed on a Netzsch Instruments NA LLC calorimeter (Burlington, MA, USA). In total, 5 mg of dried ground sample was accurately weighed in an aluminum pan. And at a rate of 10 °C/min, the sample was heated from 25 °C to 250 °C. All measurements were conducted in triplicate.

### 2.12. Solid-State ^13^C NMR Spectroscopy

All samples were dried under ambient conditions and subsequently characterized by solid-state ^13^C NMR spectroscopy using a JEOL ECZ600R 600 MHz spectrometer operating at room temperature. Spectra were acquired with a ^13^C resonance frequency of 150.87 MHz and a 90° pulse width of 2.4 μs. To maximize spectral resolution, magic-angle spinning (MAS) was employed at a rotation frequency of 15 kHz.

### 2.13. Statistical Analysis

All experiments were conducted in triplicate, and the results are presented as mean ± standard deviation (SD). Statistical analyses were performed using SPSS 7.0 software, with one-way analysis of variance (ANOVA) followed by Duncan’s multiple range test.

## 3. Results and Discussion

### 3.1. Identification of Maize Amylose and SHMAM

**Figure 1:**
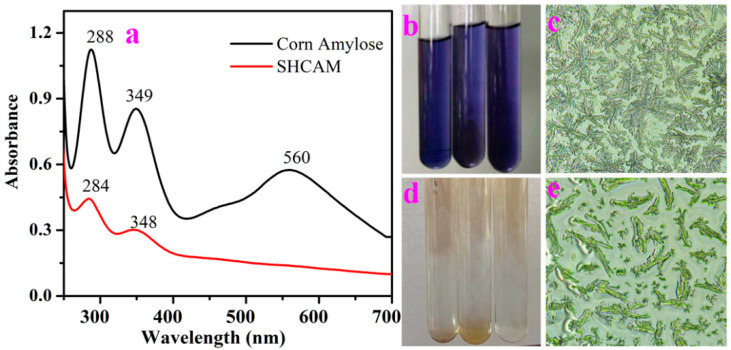
UV-Vis spectra (**a**); iodine staining color chart and optical microscopic images (100×) of maize amylose (**b**,**c**); and SHMAM (**d**,**e**).

As shown in Figure 1a, the maize amylose sample prepared via the retrogradation method exhibited characteristic iodine absorption peaks at 288 nm and 349 nm. Additionally, a distinct absorption band attributable to the amylose–iodine complex was observed at 560 nm, consistent with the well-documented deep blue coloration of this complex under ambient conditions (Figure 1b) [34]. Optical microscopic analysis revealed that the native maize amylose consisted of aggregated linear chains organized into a characteristic “willow branch” morphology, with short side chains localized predominantly at the chain termini (Figure 1c) [35]. In contrast, the single-helical maize amylose (SHMAM), synthesized by co-crystallization of maize amylose in a 0.5% (*w*/*v*) NaCl solution at 4 °C, displayed iodine-associated absorption maxima at 284 nm and 348 nm. Notably, no absorbance feature corresponding to the amylose–iodine complex was detected in the visible region (400–700 nm), and the SHMAM–iodine mixture remained colorless—lacking the diagnostic blue hue (Figure 1d). This absence strongly supports the adoption of a stable single-helical conformation, which precludes iodine encapsulation within a helical cavity. Consistent with this interpretation, high-resolution microstructural imaging (Figure 1e) confirmed that SHMAM adopts an elongated, rod-like architecture with negligible branching—exhibiting morphological features typical of low-molecular-weight, structurally constrained polysaccharides, in marked contrast to the branched, aggregated topology of native amylose.

Table S1 presents the chain length distribution characteristics of maize amylose and SHMAM. Maize amylose contained a remarkably high proportion (70.11%) of chains consisting of a single glucose residue. After multiple freeze–thaw and co-crystallization cycles, SHMAM underwent complete elimination of short-branched glucosyl units and was fragmented into low-molecular-weight species. Upon incorporation into wheat-based products, these fragments graft onto native starch chains, thereby extending the average chain length [36]. This structural modification contributes to the reduced in vitro digestibility, enhanced elastic modulus, and increased textural hardness of the final food product.

### 3.2. Influence of SHMAM on the Hardness of Steamed Buns

Figure S1 presents images of steamed buns (control sample), and steamed buns with the addition of SHMAM, stored under refrigeration for periods ranging from 0 to 6 days. The control sample appeared relatively plump and had a soft texture. As refrigeration time increased, the steamed buns gradually hardened and, by the sixth day, noticeable indentations had formed. In steamed buns with SHMAM, increased SHMAM content also led to gradual hardening. Notably, the sample with 5% SHMAM exhibited distinct surface cracking, indicating a significant influence of SHMAM on the morphology of the steamed buns. In contrast, the sample with 10% SHMAM displayed a more regular, rounded shape with fewer cracks. However, with prolonged refrigeration, all SHMAM-containing samples also underwent progressive firming and developed extensive fissures. This deterioration in texture is primarily attributed to moisture loss and the retrogradation of starch components.

As presented in Table 1, the incorporation of SHMAM exerts a significant and dose-dependent influence on the textural hardness of steamed buns. The control sample—prepared exclusively with high-gluten wheat flour—exhibited an initial hardness of 2318.7 g. In freshly prepared samples, increasing SHMAM concentration from 1% to 10% (*w*/*w*, relative to flour) induced a non-monotonic variation in hardness; hardness first increased, peaked at 5% SHMAM (3224.7 ± 98.1 g), and subsequently declined markedly at 10% SHMAM (2091.9 ± 2.6 g). These findings suggest that moderate SHMAM supplementation (e.g., 5%) enhances structural rigidity, likely through dual mechanisms: (i) restriction of water mobility within the starch–gluten interfacial region, thereby reinforcing the composite network; and (ii) modulation of molecular interactions between SHMAM and key flour constituents (e.g., gluten proteins and starch granules). Notably, all SHMAM-supplemented samples exhibited accelerated hardness increase during storage, consistent with progressive starch retrogradation [37]. Conversely, the reduced hardness observed at the highest SHMAM level (10%) may reflect excessive water sequestration by SHMAM-associated starch, thereby limiting hydration-dependent gluten network formation [22]. Further mechanistic studies—including rheological profiling, moisture distribution mapping, and microstructural analysis—are warranted to elucidate the precise structure–function relationships governing SHMAM-mediated textural modulation.

**Table 1:** Impacts of SHMAM addition on the hardness (g) of steamed buns.

Cold Storage Times (d)	Additive Amount (%)			
	0 (Control)	1	5	10
0	2318.7 ± 157.4	2752.3 ± 11.3	3224.7 ± 98.1 *	2091.9 ± 2.6
1	1233.8 ± 48.7	5156.0 ± 181.4 **	4408.2 ± 240.2 **	5398.7 ± 50.7 **
3	951.7 ± 104.8	6772.0 ± 45.9 **	6435.0 ± 266.0 **	7096.7 ± 104.6 **
6	1211.1 ± 26.8	7776.8 ± 448.3 **	8585.3 ± 24.8 **	9108.2 ± 5.3 **

* *p* < 0.05 vs. control group; ** *p* < 0.01 vs. control group.

### 3.3. Identification of SHMAM, Steamed Buns, and Steamed Buns with SHMAM

As shown in Table 2, size-exclusion chromatography (SEC) analysis revealed that maize amylose and single-helical maize amylose (SHMAM) exhibited number-average molecular weights (M_n_) of 1883 g/mol and 1675 g/mol, respectively. The lower M_n_ of SHMAM relative to native maize amylose is consistent with the partial depolymerization and/or structural rearrangement associated with single-helix formation. Both samples displayed comparable polydispersity indices (PDIs) of 4.14 and 4.20, indicating a broadly similar breadth of molecular weight distribution, albeit with a marginally broader dispersity observed for SHMAM. SEC profiles of both the control steamed bun and the steamed bun supplemented with 1% SHMAM resolved into two well-defined molecular fractions. In the control sample, these comprised a minor high-molecular-weight fraction (M_n_ = 446,483 g/mol; 12.24% area) and a dominant low-molecular-weight fraction (M_n_ = 1201 g/mol; 87.76% area). In contrast, the 1% SHMAM-supplemented sample showed a further reduced proportion of the high-molecular-weight fraction (6.24%; M_n_ = 433,250 g/mol) and a correspondingly increased proportion of the low-molecular-weight fraction (93.76%; Mn = 1168 g/mol). These results suggest that the high-M_n_ fraction likely originates from starch–protein aggregates or residual macromolecular complexes, whereas the low-M_n_ fraction predominantly represents degraded polysaccharides and small soluble carbohydrates. Notably, the PDIs of the high- and low-M_n_ fractions were 4.93 and 5.92 in the control, versus 5.06 and 3.88 in the 1% SHMAM sample—indicating that SHMAM incorporation selectively narrows the dispersity of the low-M_n_ fraction while exerting a minimal effect on the high-M_n_ fraction.

In contrast, the sample containing 5 wt% SHMAM displayed a unimodal molecular weight distribution. Its number-average molecular weight (M_n_ = 1389 g/mol) closely matched that of the low-molecular-weight fraction observed in the control sample; however, its weight-average molecular weight (M_w_ = 75,057 g/mol) was markedly elevated, yielding a polydispersity index (PDI = M_w_/M_n_) of 54.04—substantially higher than that of all other samples. The PDI represents gluten proteins, starch, etc., and this exceptionally high PDI reflects the pronounced heterogeneity in the chain length distribution of protein or starch. SEC methodology cannot distinguish them. Collectively, these findings indicate that incorporation of 5 wt% SHMAM induces substantial structural reorganization within the steamed bun matrix, fundamentally altering its macromolecular architecture [30].

### 3.4. Secondary Structures of SHMAM, Steamed Buns, and Steamed Buns with SHMAM

Table 3 summarizes the secondary structural composition (in %) of proteins associated with maize amylose and SHMAM in steamed buns. As indicated in Table 3, β-sheets constitute the predominant secondary structural element in both maize amylose–protein and SHMAM–protein complexes, accounting for 99.39% and 97.75% of the total secondary structure, respectively. In contrast, α-helical content was below the detection limit in both samples. Maize amylose-bound proteins exhibited minimal contributions from β-turns (0.54%) and random coils (0.07%), whereas SHMAM-bound proteins showed modestly elevated proportions of β-turns (0.97%) and random coils (1.27%). This increase in non-regular secondary structural elements suggests a partial loosening of protein conformation upon binding to SHMAM, consistent with findings reported by Han et al. [38].

Upon incorporation of 5% SHMAM into the steamed bun dough, the α-helix content increases modestly from 2.81% to 4.75%, whereas the β-turn content exhibits a marked increase—from 15.72% to 28.56%. In contrast, the β-sheet content decreases significantly, from 81.14% to 66.68%. These alterations in secondary structure suggest that SHMAM interacts with endogenous wheat proteins during dough mixing and subsequent steaming, thereby inducing conformational rearrangements. At a higher SHMAM concentration (10%), the α-helix, β-sheet, and β-turn contents were 6.63%, 65.38%, and 27.99%, respectively—indicating a dose-dependent shift in protein conformation, characterized by progressive conversion of β-sheets to α-helices.

The most significant conformational transition—from β-sheet to β-turn—is observed in steamed buns supplemented with 5% SHMAM. Notably, increasing the SHMAM concentration to 10% led to a marginal reduction in β-turn content. Overall, the incorporation of SHMAM elevated both α-helix and β-turn proportions, which is ascribed to specific molecular interactions between SHMAM and gluten proteins. These interactions are presumed to promote tighter aggregation and enhanced cross-linking between glutenin subunits and starch molecules, thereby facilitating the formation of a denser, more mechanically robust gluten–starch network—ultimately contributing to increased product hardness. Moreover, the complete absence of random coil structures at both 5% and 10% SHMAM levels suggests a marked reduction in unstructured protein regions available for hydration, resulting in diminished water-holding capacity. This reduced hydration capacity further reinforces the observed increase in textural firmness.

Given that the 5% SHMAM incorporation level induced the most pronounced alterations in both the secondary structure and textural hardness of the steamed bun, all subsequent structural and functional characterizations were conducted exclusively on samples formulated with 5% SHMAM. Unfortified steamed buns (i.e., containing 0% SHMAM) were employed as the control.

### 3.5. FT-IR Spectra of Maize Amylose and Steamed Buns

**Figure 2:**
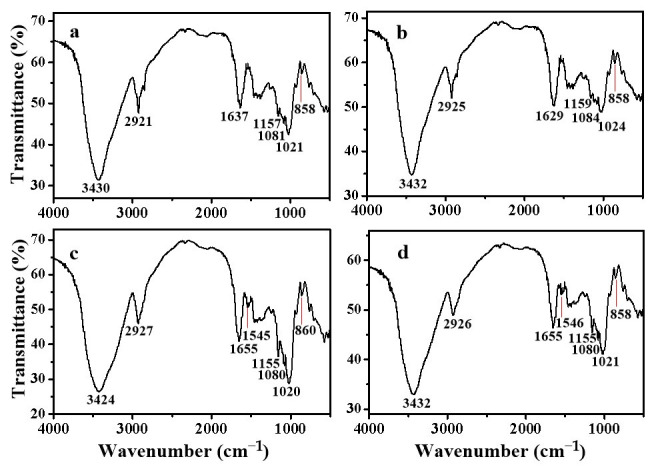
IR spectra of maize amylose and steamed bun: (**a**) maize amylose; (**b**) SHMAM; (**c**) steamed bun (control); and (**d**) steamed bun + 5% SHMAM.

Figure 2 presents the Fourier-transform infrared (FT-IR) spectra of maize amylose and steamed buns. As shown in Figure 2a, a broad absorption band centered at approximately 3230 cm^−1^ is attributed to the O–H stretching vibration of maize amylose, consistent with previously reported spectral features [39]. In contrast, the corresponding hydroxyl band in the steamed bun spectrum (Figure 2b) exhibits a slight shift to a higher wavenumber (3232 cm^−1^), suggesting a partial weakening of intermolecular hydrogen bonding—likely reflecting a structural transition toward a more flexible or less densely packed single-helical conformation.

In Figure 2c,d, the broad absorption bands centered near 3430 cm^−1^ originate from the overlapping O–H and N–H stretching vibrations. Notably, the band in Figure 2d is observed at 3432 cm^−1^, representing a hypsochromic shift of 8 cm^−1^ relative to the corresponding band in Figure 2c (3424 cm^−1^). This shift indicates a reduction in hydrogen bond strength between starch and gluten components. Nevertheless, the retention of a pronounced, broad absorbance in this region suggests the emergence of alternative intermolecular interactions. Specifically, spectroscopic evidence supports the participation of SHMAM in hydrogen bonding with native constituents of the steamed bun matrix.

Additionally, the weak absorption feature near 860 cm^−1^—widely recognized as a sensitive probe of anomeric configuration in glycosidic linkages [40]—exhibits a bathochromic shift to 858 cm^−1^ upon incorporation of SHMAM into native amylose. Concurrently, its normalized transmission intensity—referenced to the C1–O–C4′ glycosidic bond vibration at ~1020 cm^−1^ [41]—is diminished. Collectively, these spectral alterations imply the formation of novel covalent or strong non-covalent linkages between SHMAM and endogenous components during steaming.

However, the precise chemical nature and structural identity of these interactions warrant further mechanistic investigation.

### 3.6. XRD Patterns of Maize Amylose and Steamed Buns

**Figure 3:**
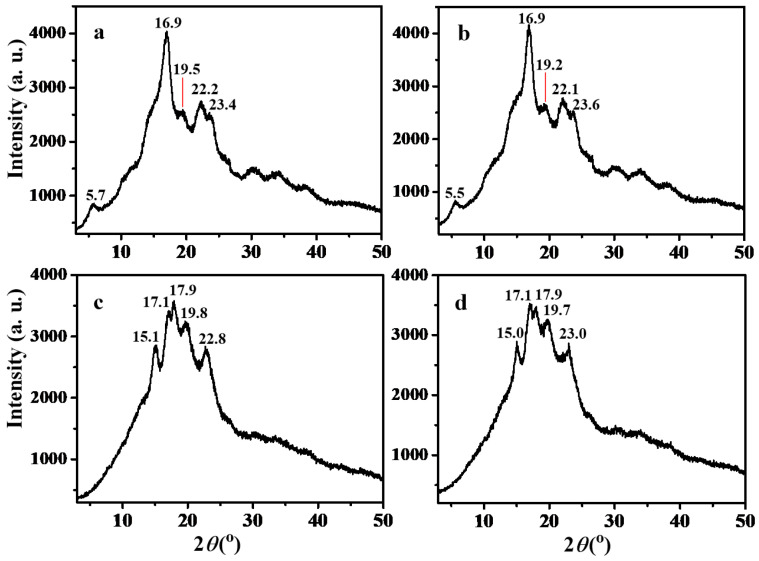
XRD spectra of maize amylose and steamed buns: (**a**) maize amylose; (**b**) SHMAM; (**c**) steamed bun (control); and (**d**) steamed bun + 5% SHMAM.

The crystalline structures of maize amylose and steamed bun samples were characterized by X-ray diffraction (XRD), as shown in Figure 3. Both maize amylose and SHMAM exhibited characteristic B-type diffraction peaks at approximately 5.5°, 16.9°, 19.5°, 22.2°, and 23.4° (Figure 3a,b). The prominent doublet near 5.5° and the strong reflection at 16.9° are diagnostic of a typical B-type crystalline polymorph, consistent with previous reports for amylose-rich starches [42]. In contrast, the control steamed bun sample displayed diffraction maxima at 15.1°, 17.1°, 17.9°, 19.8°, and 22.8°. The resolved doublet at 17.1° and 17.9° is a hallmark of the A-type crystal structure, commonly observed in native cereal starches [43].

Incorporation of 5% SHMAM into the steamed bun formulation yields an XRD pattern nearly identical to that of the control sample, indicating no phase transformation in the starch crystalline structure. However, a distinct inversion in the relative intensities of the doublet peaks at 17.1° and 17.9° is observed—specifically, the peak at 17.1° became more intense than that at 17.9°, contrary to the control. This intensity reversal suggests a microstructural modification induced by SHMAM. The 17.1° peak is probably attributable to the recrystallization of SHMAM, whereas the 17.9° peak arises from the A-type crystalline lattice of amylopectin within intact starch granules. Consistent with the prior literature [44], in A-type starches, the lower-angle shoulder (~17°) predominantly reflects amylose-associated ordering, while the higher-angle shoulder (~18°) corresponds to amylopectin crystallinity. The mechanistic basis for this intensity inversion requires further systematic investigation.

### 3.7. DSC Thermograms of Maize Amylose and Steamed Buns

**Figure 4:**
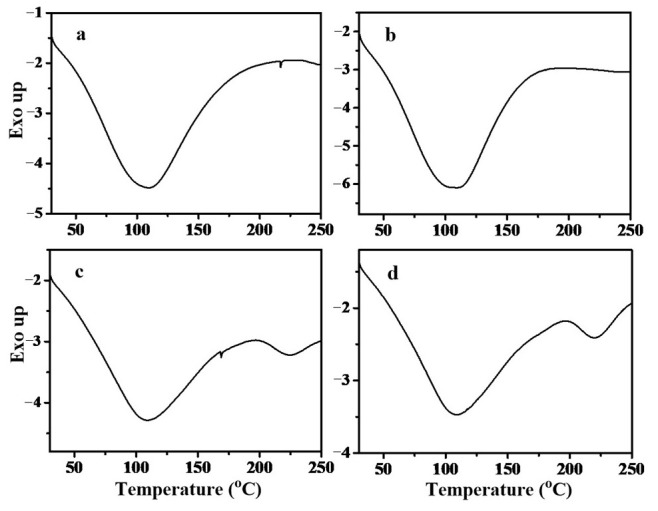
Thermal properties of maize amylose and steamed bun: (**a**) maize amylose; (**b**) SHMAM; (**c**) steamed bun (control); and (**d**) steamed bun + 5% SHMAM.

The thermal behavior of maize amylose and salt-hybridized maize amylose (SHMAM) was systematically characterized by differential scanning calorimetry (DSC), with representative thermograms and quantitative thermal parameters summarized in Figure 4 and Table 4, respectively. As established in the prior literature [45], the melting enthalpy (Δ*H*) quantifies the energy required to disrupt the ordered crystalline domains within recrystallized starch chains; thus, a higher Δ*H* value reflects greater thermal stability and structural ordering of the crystalline phase [46]. Both samples exhibited a single endothermic peak, consistent with the dissociation of amylose–lipid complexes. Specifically, maize amylose displayed a peak melting temperature (*T_p_*) of 109.00 °C and a Δ*H* of 205.92 J·g^−1^, whereas SHMAM exhibited a slightly lower *T_p_* of 105.92 °C but a significantly higher Δ*H* of 249.05 J·g^−1^. This pronounced increase in Δ*H* for SHMAM indicates that the single-helical crystalline structure formed via co-crystallization with inorganic salts exhibits enhanced molecular order and greater thermal resilience relative to native maize amylose—likely attributable to a more homogeneous chain packing and reduced structural defects within the crystalline lattice.

In contrast, two distinct endothermic transitions were observed in both the control steamed bun and the sample incorporating 5% SHMAM. The first transition, attributed to the dissociation of amylose–lipid complexes, occurred at a higher onset temperature and exhibited greater enthalpy in the control sample (*T*_*p*1_ = 108.00 °C, Δ*H*_1_ = 148.04 J·g^−1^) compared with the SHMAM-containing sample (*T*_*p*1_ = 107.04 °C, Δ*H*_1_ = 128.12 J·g^−1^). This reduction in both peak temperature and enthalpy suggests that SHMAM incorporation attenuates either the thermal stability or the extent of amylose–lipid complex formation during steaming.

The second endothermic transition—observed at substantially higher temperatures—was tentatively assigned to the melting of amylose crystallites, consistent with the prior literature [37]. In this case, the SHMAM-containing sample exhibited a marginally lower peak temperature (*T_p_*_2_ = 221.04 °C vs. 224.52 °C in the control) but a markedly higher enthalpy (Δ*H*_2_ = 8.25 J·g^−1^ vs. 4.14 J·g^−1^ in the control). This apparent inverse correlation—i.e., decreased melting temperature accompanied by increased melting enthalpy—implies that while amylose crystallites in the SHMAM sample initiate melting at a slightly reduced temperature, they possess a higher degree of structural order or crystallinity, thereby requiring greater energy input for complete disruption. We hypothesize that this enhanced molecular organization arises from specific interactions between SHMAM and gluten proteins. Supporting this interpretation, Kong et al. [47] demonstrated that low-molecular-weight proteins can insert into the helical cavity of amylose to form thermodynamically stable inclusion complexes. It is therefore plausible that SHMAM interacts analogously with glutenins, facilitating the assembly of more thermally robust and highly ordered microcrystalline domains within the steamed bun matrix.

### 3.8. Solid-State ^13^C NMR Spectra of Maize Amylose and Steamed Buns

**Figure 5:**
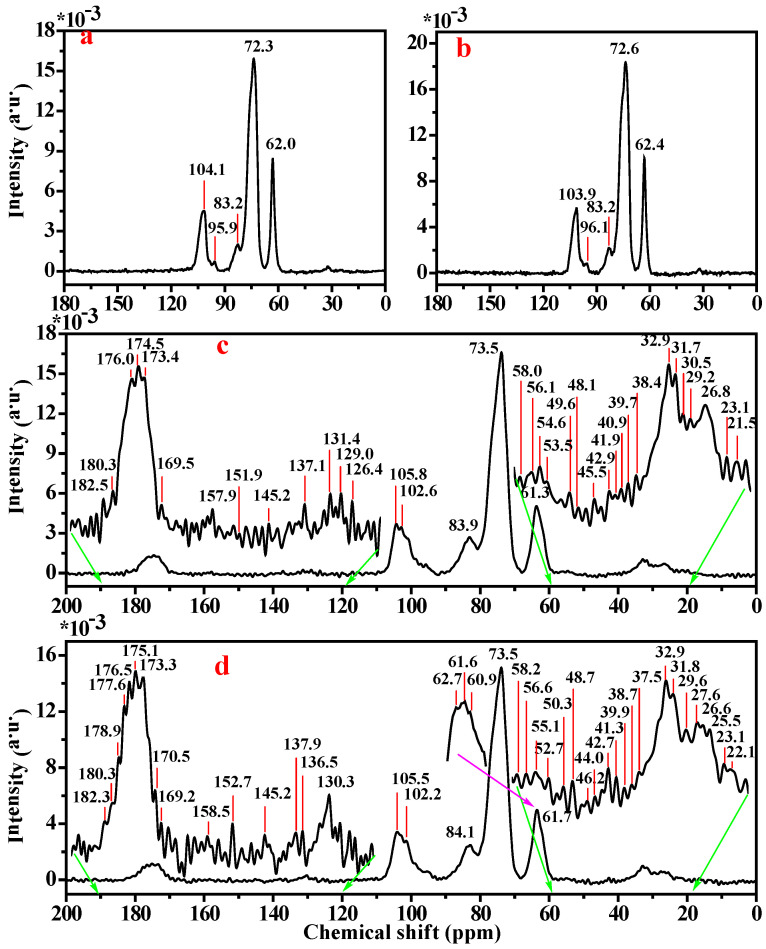
Solid-state ^13^C NMR of maize amylose and steamed buns: (**a**) maize amylose; (**b**) SHMAM; (**c**) steamed bun (control); and (**d**) steamed bun + 5% SHMAM.

As illustrated in Figure 5, the double-helical amylose structure (Figure 5a) displayed characteristic ^13^C NMR signal peaks at 104.1, 83.2, 72.3, and 62.0 ppm, assignable to C_1_, C_4_, C_2_/C_3_/C_5_, and C_6_ carbon atoms, respectively [48]. In contrast, SHMAM (Figure 5b) exhibited an upfield shift in the C_1_ resonance to 103.9 ppm, while the resonances for C_2_/C_3_/C_5_ and C_6_ underwent slight downfield shifts to 72.6 ppm and 62.4 ppm, respectively. These chemical shift changes indicate an increase in electron density at C_1_ and a concomitant decrease at C_2_/C_3_/C_5_ and C6 upon transition from the double-helical to the single-helical conformation. The upfield shift in C_1_ is consistent with reduced involvement in intermolecular hydrogen bonding—likely due to partial detachment from the interchain H-bond network—accompanied by increased shielding arising from proximity to adjacent glucose units and enhanced participation in intramolecular hydrogen bonding, which collectively stabilize the glycosidic linkage geometry. Concurrently, the downfield shifts observed for C_2_/C_3_/C_5_ and C_6_ reflect diminished electron density, attributable to partial disruption of intermolecular hydrogen bonds involving the C_2_–OH and C_3_–OH groups, coupled with formation of more compact, stabilizing intramolecular hydrogen bonds. Notably, the release of C_6_ from the steric and electronic constraints imposed by the double-helical packing allows for its hydroxymethyl group to engage more readily in the intramolecular hydrogen-bonding network of the single helix, thereby increasing its hydrogen-bonding occupancy and resulting in the observed downfield displacement.

The steamed bun sample in the absence of SHMAM (Figure 5c) displayed two distinct C_1_ signal resonances at 105.8 and 102.6 ppm, alongside characteristic signals at 83.5, 73.5, and 61.3 ppm, assignable to C_4_, C_2_/C_3_/C_5_, and C_6_, respectively. Upon incorporation of SHMAM (Figure 5d), the C_1_ resonances underwent an upfield shift to 105.5 and 102.2 ppm, whereas the C_4_ signal shifted downfield to 84.1 ppm. Most significantly, the C_6_ resonance resolved into three well-separated peaks at 62.7, 61.6, and 60.9 ppm. These spectral changes suggest that the C_1_ carbon of SHMAM is positioned within the shielded interior of the helical cavity, while C_4_ resides in the deshielded exterior region. The pronounced triplet splitting observed for C_6_ not only reflects substantial reorganization of the hydrogen bonding network but also implies the emergence of additional site-specific chemical interactions—potentially including altered hydration, conformational constraints, or non-covalent binding effects—at this position.

In steamed buns supplemented with 5% SHMAM, cysteine—the β-turn-characteristic amino acid integral to the secondary structure of glutenin—exhibits a downfield shift in the chemical shift in its β-carbon resonance from 29.2 ppm to 29.6 ppm, indicative of hydrogen bond formation involving the sulfhydryl group at this residue. Likewise, the α-carbon signals of lysine undergo a downfield shift from 56.1 ppm to 56.5 ppm, consistent with hydrogen bonding at these sites. Furthermore, the carbon atom bearing the phenolic hydroxyl group of tyrosine and the ε-carbon of arginine both show a downfield shift from 157.9 ppm to 158.5 ppm, collectively supporting enhanced hydrogen bonding interactions at these residues.

**Figure 6:**
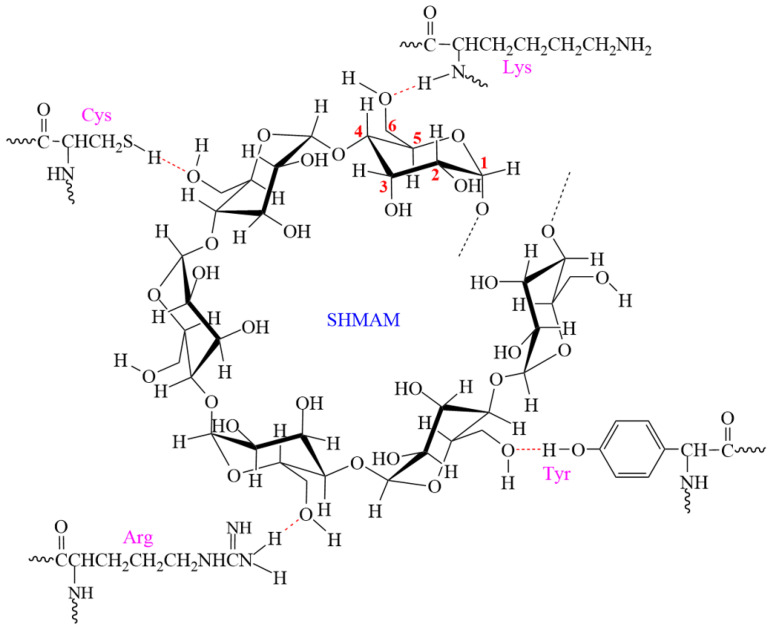
The interaction of SHMAM with the steamed bun.

Spectroscopic analysis of SHMAM reveals that specific amino acid side chains in glutenin—namely, the sulfhydryl group of cysteine, the α-amino group of lysine, the phenolic hydroxyl group of tyrosine, and the ε-amino group of arginine—form hydrogen bonds with the C_6_ hydroxyl groups of SHMAM molecules (Figure 6). These non-covalent interactions collectively occupy hydration sites associated with glutenin’s hydrophilic functional groups, thereby diminishing its water binding capacity. Concurrently, enhanced molecular embedding of starch granules within the modified glutenin network increases structural stability, which suppresses starch granule swelling and ultimately contributes to increased hardness in flour-based products.

### 3.9. Mechanism of SHMAM-Induced Hardness Enhancement in Steamed Buns

**Figure 7:**
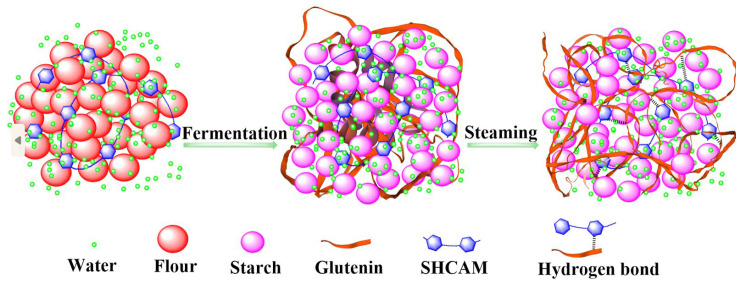
The mechanism of SHMAM-induced hardening of steamed bread.

The mechanism underlying SHMAM-induced enhancement of steamed bread hardness is delineated as follows (Figure 7). During steaming, SHMAM engages in specific molecular interactions with glutenin subunits—namely, hydrogen bond formation—with selected amino acid residues. These interactions trigger a pronounced conformational rearrangement in the glutenin secondary structure, marked by a reduction in β-sheet content and a concurrent increase in α-helical and β-turn conformations. As a result, intermolecular aggregation and cross-linking between glutenins and starch are significantly augmented, facilitating the formation of a denser, more cohesive macromolecular network. Concurrently, SHMAM restricts translational and rotational mobility of interfacial water molecules at the starch–glutenin interface. Moreover, SHMAM competitively occupies hydration sites on the hydrophilic side chains of glutenins, thereby attenuating their intrinsic water holding capacity. Collectively, these covalent and non-covalent interactions synergistically promote network densification, enabling more uniform and stable embedding of starch granules within the glutenin matrix. This refined structural architecture effectively suppresses excessive starch granule swelling during gelatinization, enhances microstructural homogeneity, and reinforces the mechanical resilience of the crumb. Consequently, both hardness and chewiness of the steamed bun are measurably improved.

## 4. Conclusions

This study employed a salt co-crystallization method to synthesize SHMAM, which was subsequently incorporated into wheat flour to prepare steamed buns. The influence of SHMAM supplementation on the hardness of the steamed buns was systematically evaluated. The 5% (*w*/*w*) incorporation of SHMAM conferred maximal hardness as SHMAM formed hydrogen bonds with specific amino acid residues in glutenin during steaming. These interactions triggered a pronounced conformational rearrangement in the secondary structure of glutenin, characterized by a reduction in β-sheet content and a corresponding increase in α-helix and β-turn conformations. Consequently, a denser and more mechanically robust glutenin–starch network was formed. This structural reorganization diminished the water holding capacity of glutenin and impeded water mobility between starch and glutenin phases. The synergistic action of non-covalent (hydrogen) interactions enhanced dough compactness, stabilized the microstructural integrity of the system, and improved the mechanical performance of the final product. Collectively, this work establishes a rational, scalable strategy for modulating steamed bun hardness through targeted macromolecular engineering of glutenin functionality—offering promising implications for texture optimization and nutritional quality improvement in staple wheat-based foods. Since there is no significant analysis of interaction between factors, the experimental results of this paper are for reference only.

## Figures and Tables

**Table 2:** The molecular weight distribution of SHMAM; steamed buns before and after the addition of SHMAM.

Items	Maize Amylose	SHMAM	Steamed Bun (Control)	Steamed Bun Coupled with SHMAM (1%)	Steamed Bun Coupled with SHMAM (5%)
Retention time (min)	18.73	18.76	15.45 19.15	15.267 19.350	19.350
M_n_ (g/mol)	1883	1675	446,483 (12.24%) 1201 (87.76%)	433,250 (6.24%) 1168 (93.76%)	1389
M_w_ (g/mol)	7805	7038	2,200,691 (12.24%) 7104 (87.76%)	2,194,215 (6.24%) 4534 (93.76%)	75,057
M_p_ (g/mol)	5579	5349	526,112 3119	678,306 2364	2364
PDI (M_w_/M_n_)	4.14	4.20	4.93/5.92	5.06/3.88	54.04

**Table 3:** The secondary structures of streamed buns combined with/without SHMAM (%).

Samples	α-Helix (%)	β-Sheet (%)	β-Turn (%)	Random Coil (%)
Maize amylose	0	99.39	0.54	0.07
SHMAM	0	97.75	0.97	1.27
Steamed bun (control)	2.81	81.14	15.72	0.32
Steamed bun + 5% SHMAM	4.75	66.68	28.56	0
Steamed bun + 10% SHMAM	6.63	65.38	27.99	0

**Table 4:** Thermal properties of maize amylose and steamed buns.

Samples	*T*_o1_ [°C]	*T*_p1_ [°C]	*T*_c1_ [°C]	Δ*H*_C1_ [J·g^−1^]	*T*_o2_ [°C]	*T*_p2_ [°C]	*T*_c2_ [°C]	Δ*H*_C2_ [J·g^−1^]
Maize amylose	47.16	109.00	172.36	205.92	-	-	-	-
SHMAM	46.79	105.92	161.29	249.05	-	-	-	-
Steamed bun (control)	62.31	108.00	173.76	148.04	204.33	224.52	244.89	4.14
Steamed bun + 5% SHMAM	48.11	107.04	176.87	128.12	201.18	221.04	243.66	8.25

## Data Availability

The original contributions presented in this study are included in the article/Supplementary Material. Further inquiries can be directed to the corresponding authors.

## Supplementary Materials

The following supporting information can be downloaded at: https://www.mdpi.com/article/10.3390/foods15101821/s1, Figure S1. Impacts of SHCAM addition on the hardness of steamed bread. Table S1. The chain length distribution of corn amylose and SHMAM.
