# Supplementary material for: Understanding the Impact of Single-Helical Maize Amylose on Steamed Bun Hardness Enhancement
Source: Foods. 2026 May 21;15(10):1821. doi: 10.3390/foods15101821 (PMC13206128; doi:10.3390/foods15101821)
Supplement: Supplementary file 1 [file foods-15-01821-s001.zip › foods-4288130-supplementary.pdf]

foods

Supplementary Materials

Understanding the Impact of Single-Helical Maize Amylose on Steamed Bun Hardness Enhancement

Jiarui Yu, Zhihui Zhang, Shuai Ran, Xiaoxiao Li, Chunrui Wang, Junjie Guo * and Xijun Lian *

Tianjin Key Laboratory of Food Biotechnology, College of Biotechnology and Food Science, Tianjin University of Commerce, Tianjin 300134, China; yjr200109@126.com (J.Y.); zhanghuizhi@tjcu.edu.cn (Z.Z.); ranshuai@tjcu.edu.cn (S.R.); lixiaoxiao@tjcu.edu.cn (X.L.); wangchunrui@tjcu.edu.cn (C.W.)
* Correspondence: gjjie@tjcu.edu.cn (J.G.); lianxijun@tjcu.edu.cn (X.L.)

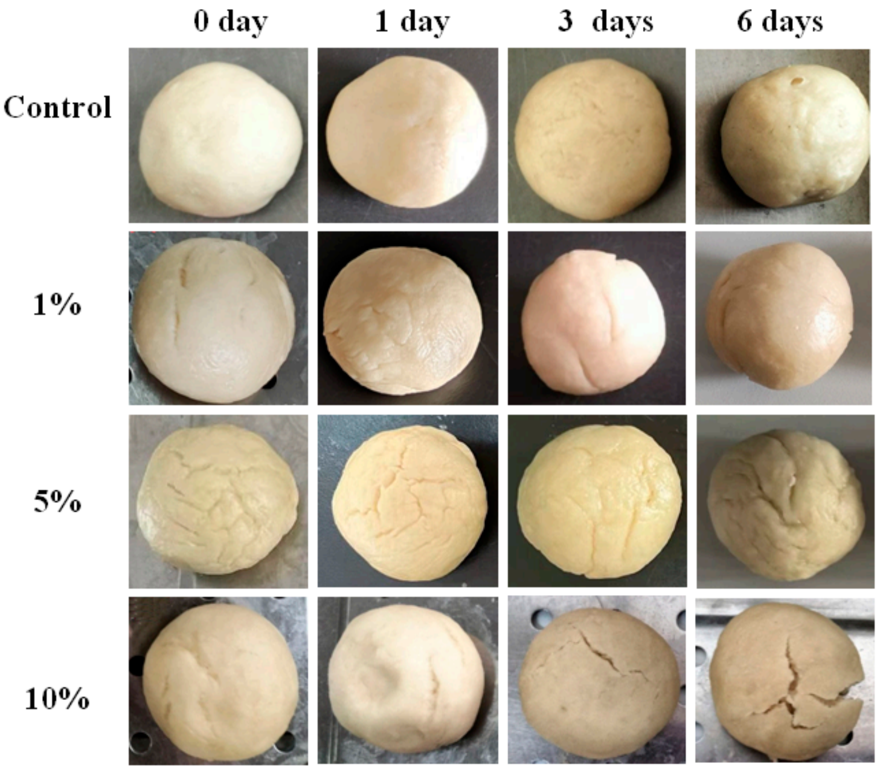

Figure S1. Impacts of SHCAM addition on the hardness of steamed bread.

Table S1. The chain length distribution of corn amylose and SHMAM.

Chain length (Glucose number)	Corn amylose	SHMAM
1	70.11	0
2	1.59	0
3	2.14	0
4	0.95	0
5	2.96	0
6	1.07	0
7	2.8	0
8	1.65	0
9	0.71	0
10	0.61	0
11	0.28	0
12	0.51	0
13	0.57	0
14	0.65	0
15	0.94	0
16	1.02	0
17	1.08	0
18	1.14	0
19	1.08	0
20	1.03	0
21	1.01	0
22	0.93	0
23	0.93	0
24	0.76	0
25	0.67	0
26	0.57	0
27	0.47	0
28	0.4	0
29	0.3	0
30	0.23	0
31	0.28	0
32	0.15	0
33	0.17	0
34	0.12	0
35	0.07	0
36	0.05	0

https://doi.org/10.3390/foods15101821
